# Remarks on Congestive Fever; with Cases

**Published:** 1846-10

**Authors:** Francis H. Gordon

**Affiliations:** Clinton College, Tennessee


ART. II.—*Remarks on Congestive Fever; with Cases.* By FRANCIS H. GORDON, M.D., of Clinton College, Tennessee.

The subject of Congestive Fever is one of so much interest to the profession in our country, that individual experience in the disease is not likely to be disregarded. . In the hope that what I have observed in the cases which have fallen under my care may be useful to others, I have drawn up the following paper. I shall enter at once upon the history of the cases, following them by such remarks as have been suggest ed by my experience.

September 15th, 1845, I was called to T. Walker, æt. about 50 years. I found him in a "congestive chill," which had already persisted four hours. His extremities were cold; he complained of insatiable thirst, with an excruciating, burning pain in the stomach, attended with retchings; he had pain in the head also, and in his loins, and limbs, and a sense of weight along the sternum, with dyspnœa; his face was purplish, and in spots livid; his abdomen tender to the touch, and hard; his alvine dejections abundant, consisting of yellow water; urine scanty and red; tongue red, dry, and rough; pulse 100 in the minute, small and corded. As is usual in such chills, the patient expected hourly to die, and when the chill finally went off he expressed the fullest conviction that he could not survive a second one.

I gave the patient immediately 25 grains of carbonate of ammonia, and a grain of acetate of morphia, and applied a towel wet with cold water to the epigastrium. The ammonia was repeated every twenty minutes, and in about an hour after the exhibition of the first dose the circulation became general and the symptoms grew less threatening. I directed 18 grains of calomel, 15 grains of Dover's powder, and 6 grains of acetate of lead, each to be divided into three equal portions, one of which the patient was to take every six hours, and, if necessary, to promote their action by castor oil in six hours after taking the first powder. I also ordered blood to be drawn to the extent of softening the pulse so soon as reaction should become complete. For the next day I gave the following prescription: Four hours before the chill is expected, give patient 20 grains carb. ammonia, and 2 grains of quinine; repeat the ammonia every half hour conjoined with spirits of nitre, ʒj., until within one hour of the chill, and then give him 2 grains of quinine and 1 grain of morphia, with ʒij. spts. nitre, and repeat the ammonia and spirits of nitre every half hour till the time for the chill has passed. About the hour of the expected access, apply sinapisms and hot irons to his feet, and keep him closely covered.

16th. At 4 o'clock, P. M., I found that the calomel had operated too freely, inducing green watery evacuations. The patient had escaped the chill, but his fever had been very high; his tongue was red and dry, and adhered to the finger; the epigastrium tender to the touch; sleep disturbed. Prescribed quinine, ammonia, and spirits of nitre as on the preceding day, to prevent the chill, adding morphia to the prescription; acetate of lead and Dover's powder, of each 2 grains, to be given often enough to reduce the alvine dejections to one aday, and if these are not sufficient to that end, use gruel enemata, containing 30 drops of laudanum, and 4 grains acetate of lead; pour a stream of warm water for four hours on the epigastrium, and follow this by hot bran poultices, to be renewed every twenty minutes.

17th. Patient a little better; had escaped his chill; with great difficulty the action of the bowels had been kept in bounds by means of the astringent and anodynes by mouth and rectum; less heat and tenderness of stomach; pulse still small, frequent and hard; abdomen swollen, hot externally, and tender to the touch; tongue as before. Directed acetate of lead, Dover's powder, and laudanum, as before; stream of warm water upon the abdomen for four hours and followed by bran poultices over the whole surface; half grain doses of morphia often enough to keep him quiet; ʒi. spts. nit. every hour till skin becomes moist.

18th. Slight general improvement. All the treatment of yesterday to be continued.

19th. Patient has had a slight chill, and symptoms are aggravated in consequence of his having had three alvine discharges in rapid succession before a speedy resort to the prescribed remedies had had time to control the bowels. Directed the stream of hot water and poultices as before; quinine, morphia and spts. nit. as in the onset to prevent the chill; 20 drops oil of turpentine thrice daily; 4 gr. ää calomel and Dover's powder to be taken instantly, and guarded, if required, by lead, laudanum, and Dover's powder, as before.

20th. Patient improved; has had but one consistent, dark green evacuation; had no chill; slept well three hours; tongue and skin not so dry; pulse less frequent and more soft. Directed 2 grs. quinine and 1 ʒ spts. nit. to be given when the fever abates, and repeat in two hours; lead, morphia, Dover's powder and poultices as before.

21st. Patient improving. Treatment continued. From this time till the 30th the treatment was but little varied, when sulph. and carb. magnesia were administered in small and repeated portions, guarded with lead and Dover's powder. Added to these I ordered as a tonic an infusion of quassia in doses of an ounce thrice daily. During the whole treatment the patient had been allowed only seven or eight teaspoonfuls of soup or panada a day; but his allowance was now gradually increased. Recovery was slow but progressive, and by the 25th October he was able with care to attend to business on the farm.

The foregoing is one case illustrative of many, which occurred to the writer during the prevalence of the disease in 1844 and '45. It is not reported with the view of presenting any unusual feature of disease, but from the impression that as much, or perhaps more good may be done by occasional reports of cases, with which the practitioner has usually to contend, than of such as are rare, and therefore less important.

I believe it is now common to attribute the phenomena of "congestive chill" or "stage of deprsssion" to two proximate causes, viz.: depression and irritation of the nerves of organic life. But along with this view, it is also common to condemn the administration of stimulants during the chill, on the alleged ground that these remedies are apt to do damage by "causing inflammation in the suffering organs," or by "producing an effect on the nervous system analogous to its then morbid state;" in other words, they will increase the morbid depression and irritation on which the chill depends. My observation is at variance with this inculcation. I could give many cases to prove, not only that stimulants do no injury in the stage of depression, but that they do considerable good, and are therefore indicated and demanded. Take one or two examples. Some time in the autumn of 1837, I went with Dr. B. W. Avent, of Rutherford county, to see some patients of his suffering with this form of fever. The object of my ride was to see his practice tested. Some of his patients had been treated on the popular plan, and others had been stimulated during the chill with large quantities of brandy, and bled to incipient syncope, as soon as there was partial reaction. Though I could hardly persuade myself to become a full convert to Dr. A.'s new practice, yet there was no question but that he had been more successful where the brandy had been used. I well recollect two patients in the same house, a brother and sister, who had been treated, the one on the popular plan, and the other with brandy; and the difference in favor of the stimulant could not be misunderstood. The Doctor had been induced to adopt the plan, because he had lost two or three patients for want of complete reaction; and he stated that a majority of those he cured narrowly escaped the grave, and then very slowly recovered. He had not yet seen any injury from the brandy.

I must be indulged in giving one case of my own. June 5th, 1844, I saw Mr. J. S., æt. about 40 years. His system had partially reacted from a chill, which much alarmed him and the family. I left him medicines to ward off two chills, consisting of 25 to 30 grs. quinine, in solution of paregoric and spts. nitre, ää ℥iss. The next chill was expected at 8 o'clock, A. M., on the 7th. I directed him to begin at 2 o'clock and take ʒi. of the liquid every half hour till 9 o'clock, and as he was partial to pepper, allowed him to drink red pepper tea as he desired. On the 7th, about 11 o'clock, A. M., I was called in haste. The messenger reported Mr. S. as in a dying condition. I found him breathing with difficulty; pulse frequent, but full and not very hard; great internal heat and thirst; whole surface hot and very red. The great dread of another chill had caused the patient to commence taking the remedies at 10 o'clock the night of the 6th, and he had continued up to 9 o'clock that morning; in short, he had taken nearly all the quinine preparation, and besides had taken not less than a quart of red pepper tea. A weak solution of nit. potash as a common drink, and frequent sponging of the surface with cold water made the patient more comfortable, and recovery was rapid without another chill.

September 20, 1845. Saw Mr. E. Rollings in second chill. Symptoms could hardly paint a more severe chill, or more intense suffering. I immediately gave him a grain and a half of acetate of morphia, 25 grs. carb. ammonia and iʒ. oil of turpentine. In about one hour the patient was easy, and I left him in a quiet sleep. It was the first case I had attended in the family, and the quaint remark of the patient the next day was, "that the Doctor had knocked the chill sky high."

Having combated many cases in the height of the chill, there is no question with the writer but that with morphia or opium, carb. ammonia and oil of turpentine, the worst chills may be moderated and shortened, and that a majority may be limited to one or two hours from the first administration of the medicines; and, instead of exciting inflammation, the tendency to it is certainly diminished by these remedies.

Having "crossed the Rubicon," in venturing to oppose the popular teaching in regard to this fever in one respect, the reader is no doubt prepared to see my presumption lead me to speak in disparagement of quinine itself—the anchor of hope in all autumnal fevers. This remedy has been so highly and so universally extolled that its eulogy is common-place, and therefore no commendation. See how extravagant is the estimate of a good remedy. It is popularly said that "quinine is stimulant, tonic, diaphoretic, and, to some extent, narcotic and sedative—is capable of setting aside the febrile diathesis, and establishing in its stead a *specific* quinine diathesis, and is therefore an alterative." We are taught that quinine is the true and only specific in autumnal fevers. We are to bleed, vomit and purge to prepare for the quinine; give diaphoretics and diuretics to prepare for the quinine; cup, leech and blister, all to prepare for the specific, quinine. And some southern practitioners do not think it necessary "to prepare for the quinine" at all, but give it largely *ab ovo usque mala.* We are taught to give it in such quantities as will insure *tinnitus aurium.*

With all deference to high authority, let facts have a hearing. During the autumn of 1844, while the form of fever above described prevailed so generally as to keep every practitioner under a press, the writer ran out of quinine, and could get none at the drug stores in Nashville, nor could any be borrowed of neighboring practitioners. All efforts to procure the article having failed, I feared that some of my patients would die for the want of that valuable medicine. But to do the best I could in that state of things, I determined to rely on oil of turpentine, carb. ammonia and morphia; and the result of three weeks' day and night practice was, that all cases, from the mildest to the most violent, appeared to be as easily and as successfully managed, as they had been by the instrumentality of quinine. During the highest rage of this fever in 1845, which was truly alarming, I was out of quinine for more than two weeks, and succeeded just as well as when I had ounces of the article on hand. When a chill has commenced, my observation proves that quinine is of no avail in arresting it; nor do I believe that that article *alone will invariably prevent a chill*, however long it may be given in anticipation. I consider opium or morphia more certain in arresting or preventing a chill than quinine; and when you prevent two chills in succession, the paroxysms are broken up, and the disease is easily managed. Any powerful stimulant, with a narcotic, given sufficiently in advance, and continued to the expected hour, will generally be effectual in warding off the chill. But the chief reliance should be upon a narcotic, of which opium is the best, to quiet or suspend the peculiar irritation on which the phenomena of the chill mainly depend. Allay the irritation, and there will be less manifestation of depression. When the regular paroxysms are broken up, I consider quinine more valuable as a tonic and permanent stimulant than in the onset of the disease. It equalizes the circulation and sustains the prostrate energies, until the system has time to recover from the shock it has sustained. But while quinine must be admitted to be a remedy of rare value, the above facts go to prove that its importance may be overrated, and that fever will still be cured after the present rage for that article shall have exterminated the whole genus Cinchona.

June, 1846.

